# BDNF expression in brain regions of Anorexia Nervosa mouse model, a biomarker of diagnostic and prognostic?

**DOI:** 10.1192/j.eurpsy.2023.766

**Published:** 2023-07-19

**Authors:** N. Ramoz, J. Cao, C. Tezenas du Montcel, V. Tolle, P. Gorwood, O. Viltart

**Affiliations:** 1 Institute of Psychiatry and Neuroscience of Paris, INSERM U1266; 2 CMME, GHU Sainte-Anne Hospital, Paris; 3 Université de Lille, Lille, France

## Abstract

**Introduction:**

Anorexia nervosa (AN) is a complex mental disorder mainly characterized by a voluntary food restriction and excessive physical activity resulting in dramatic weight loss. Changes in the brain-derived neurotropic factor (BDNF) have been reported in AN patients compared to controls. According to meta-analysis, functional variant rs6265 Val66Met of the *BDNF* gene has been found genetically associated to AN. We also reported an association of this functional variant and electrodermal response to images of thinness suggesting an association between rs6265 and a reward effect of weight loss in AN. In animal models, BDNF modulates negatively the central control of food intake and its injection in rodents induces weight loss and anorexia. Thus, besides its function on neuronal survival, synaptic plasticity and mood, BDNF was also reported to have a metabolic effect via both central nervous system and peripheral organs, which makes BDNF a good candidate for AN diagnosis biomarker.

**Objectives:**

Our study investigates the levels of expression of Bdnf, gene and protein, taking advantage of the mouse AN-like model by measuring Bdnf levels in specific brain areas and blood in food-restricted and refeed animals.

**Methods:**

We used a mouse AN-like model combining a phase of chronic food restriction (50%) during 15 days followed by an *ad libitum* refeeding period of one week. Female mice have or not access to a running with wheel to create a similar metabolic environment that those patients suffering from AN during restriction and recovery once hospitalised. The Bdnf mRNA and protein levels were measured in samples of blood and brain regions (prefrontal cortex, hippocampus, hypothalamus, dorsal striatum, nucleus Accumbens, ventral tegmental area and amygdala) using quantitative PCR and ELISA methods in the different groups of mice (ad libitum, ad libitum with wheel, food restriction and food restriction with wheel). Statistical analysis will compare the measures for different samples by one-way or two-way ANOVAs depending the group of animals or brain regions and blood.

**Results:**

To date, no difference of the level of transcription for *Bdnf* was observed between the different groups of mice (ad libitum, ad libitum with wheel, food restriction and food restriction with wheel) in the prefrontal cortex, hippocampus and hypothalamus. We expect significant differences of Bdnf expression in the other brain regions of interest for the food restricted animals with or without the wheel compared to ad libitum animals. We expect also differences in the level of expression of Bdnf in fasted animals compared to the refeed animals.

**Conclusions:**

The BDNF could represent a potential biomarker of AN for the diagnostic and the prognosis in the evolution to the remission when weight recover and thus will allow a better understanding of the aetiology of AN. This study is supported by Fédération pour la Recherche sur le Cerveau.

**Disclosure of Interest:**

None Declared

